# Detailed methylation map of LINE‐1 5′‐promoter region reveals hypomethylated CpG hotspots associated with tumor tissue specificity

**DOI:** 10.1002/mgg3.601

**Published:** 2019-04-06

**Authors:** Amit Sharma, Muhammad A. Jamil, Nicole Nuesgen, Albertas Dauksa, Antanas Gulbinas, Wolfgang A. Schulz, Johannes Oldenburg, Osman El‐Maarri

**Affiliations:** ^1^ Institute of Experimental Hematology and Transfusion Medicine Bonn Germany; ^2^ Department of Neurology University Clinic Bonn Bonn Germany; ^3^ Institute for Digestive Research Lithuanian University of Health Sciences Kaunas Lithuania; ^4^ Department of Urology Medical Faculty Heinrich‐Heine‐University Düsseldorf Germany; ^5^ Department of Natural Sciences Lebanese American University Beirut Lebanon

**Keywords:** biomarker, bisulfite modification, cancer, DNA methylation, epigenetics, LINE‐1, methylation patterns, SIRPH, tumor marker

## Abstract

**Background:**

Long interspersed nuclear elements (LINE‐1) sequences constitute a substantial portion of the human genome, and their methylation often correlating with global genomic methylation. Previous studies have highlighted the feasibility of using LINE‐1 methylation to discriminate tumors from healthy tissues. However, most studies are based on only a few specific LINE‐1 CpG sites.

**Methods:**

Herein, we have performed a systematic fine‐scale analysis of methylation at 14 CpGs located in the 5′‐region of consensus LINE‐1, in bladder, colon, prostate, and gastric tumor tissues using a global degenerate approach.

**Results:**

Our results reveal variable methylation levels between different CpGs, as well as some tissue‐specific differences. Trends toward hypomethylation were observed in all tumors types to certain degrees, showing statistically significance in bladder and prostate tumors. Our data points toward the presence of unique LINE‐1 DNA methylation patterns for each tumor type and tissue, indicating that not the same CpGs will be informative for testing in all tumor types.

**Conclusion:**

This study provides an accurate guide that will help to design further assays that could avoid artifacts and explain the variability of obtained LINE‐1 methylation values between different studies.

## 1 BACKGROUND

Long interspersed nuclear elements (LINE‐1 [L1]) are the most abundant retrotransposon sequences occupying approximately 17% of the human genome (Lander et al., 2001). Although the majority of L1 elements are truncated, mutated and/or have acquired deletions/insertions at their 5′‐end sequence and have therefore no longer the ability to transpose, a relatively small number in human remain retrotransposition competent (Brouha et al., 2003). Although LINE‐1 elements are typically heavily methylated in normal tissues, LINE‐1 hypomethylation has been reported in many tumor types (Antelo et al., 2012; Choi et al., 2007; Gao et al., 2014; van Hoesel et al., 2012) including bladder (Jurgens, Schmitz‐Drager, & Schulz, 1996; Wilhelm et al., 2010; Wolff et al., 2010), colon (Ogino et al., 2008), prostate (Florl et al., 2004), and stomach (Shigaki et al., 2013). LINE‐1 hypomethylation might therefore serve as a cancer biomarker. However, its extent and pattern has proved to be variable and not equally rewarding among different tumors. For instance, in a comparison of multiple cancer types, Nüsgen et al. (2015) observed that tumors could be ranked by their LINE‐1 hypomethylation levels with urinary bladder cancers being most hypomethylated followed by prostate, colon, and stomach cancers. The mechanism underlying the preferential hypomethylation in certain cancer types are still obscure.

Although LINE‐1 methylation generally correlates with global DNA methylation, and thus reflects the cellular status, an obstacle to using LINE‐1 methylation as a biomarker is the weak reproducibility between different studies (Choi et al., 2009; Ohka et al., 2011). One reason for this could be the use of different assays targeting different CpGs within LINE‐1 by different labs. Therefore, in this study, we determined the methylation levels of all technically accessible 14 different CpG sites within the 5′‐region of consensus LINE‐1 (L1Hs) using a degenerate amplification approach with a quantitative methylation‐dependent primer extension assay (SIRPH) protocol.

## 2 MATERIALS AND METHODS

For methylation analysis, tissues from bladder (15 tumor: 11 paired healthy), colon (13 tumor: 11 paired healthy), stomach (12 tumor: 9 paired healthy), prostate (21 tumor: 15 paired healthy), and blood (healthy: 33) were included. These samples are described in detail in Ref. 13. DNA methylation levels in LINE‐1 repetitive sequences were determined by using modified version of methylation‐dependent primer extension assays (SIRPH) based on HPLC as previously described by El‐Maarri, (2004) and El‐Maarri, Herbiniaux, Walter, & Oldenburg, (2002). Since the multiplexing capacity of this method is up to three CpGs sites, we included in every run the CpG 7 site assay primers as internal control to normalize the methylation levels of different CpG sites within the same sample DNA. Statistical analysis of differential methylation was performed with Prism software (GraphPad Prism version 5.0f, GraphPad Software, San Diego, CA, www.graphpad.com).

## 3 RESULTS AND DISCUSSIONS

**Figure 1:**
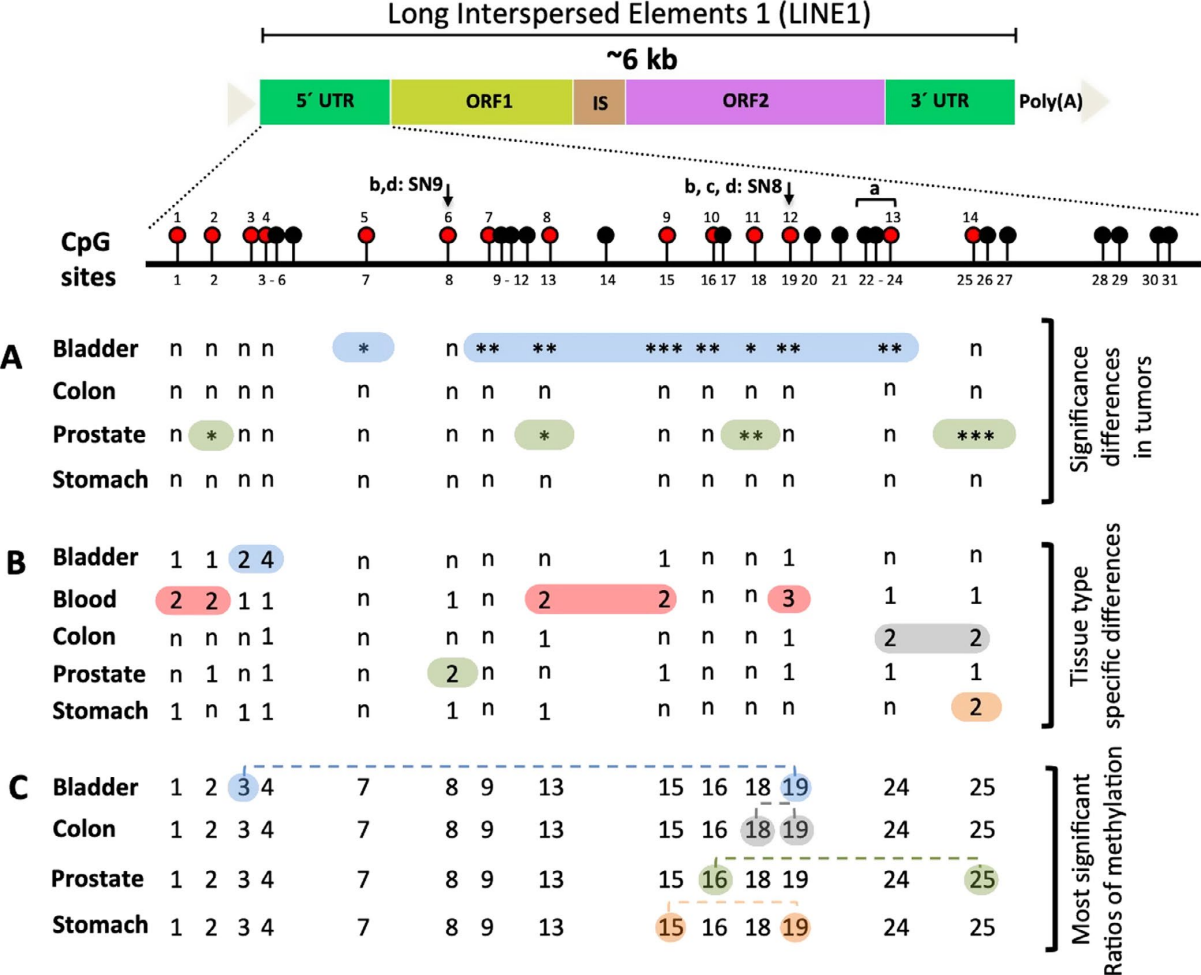
Graphical summary of results obtained in this study. The upper part shows a schematic diagram of the LINE‐1 sequence with the CpGs consecutively numbered. Red balls represent the CpG sites investigated in this study. Vertical arrows represent SN9 and SN8 as studied by El‐Maarri et al. (2007) (b) and El‐Maarri et al. (2011) (c), and Nüsgen et al. (2015) (d); the bracket labeled “a” indicates the CpG‐sites covered by the PyroMark LINE‐1 kit (Qiagen, Hilden, Germany). (A) Summary of significant differences between tumor and healthy neighboring tissue (*,**, and *** correspond to significant values of *p* < 0.05, <0.005, and <0.0005); n: nonsignificant; based on data in Figure S1). (B) CpGs with healthy tissue‐specific significance are labeled. The numbers indicate the count of significant differences in methylation levels between any two tissues (n: nonsignificant; based on data in Figure S2). (C) The ratio of CpGs methylation that is most significantly different between tumor tissues and controls are connected by dashed line (based on data in Figure S3). LINE‐1: long interspersed nuclear elements‐1

In order to address the above‐mentioned issues complicating the investigation of LINE‐1 methylation, we designed a PCR‐based degenerate assay to analyze the complete cluster of CpG dinucleotides in the consensus LINE‐1 5′‐region, in order to identify methylation changes at LINE‐1 promoter region in various carcinoma types (bladder, colon, prostate, and stomach). In this cluster, 31 individual CpG sites were identified in a region stretching across approximately 500 bp (L1Hs consensus sequence; NCBI accession number: X580759; Figure 1). SIRPH targeting primers could be designed to examine the methylation levels at 14 CpG sites (CpGs: 1–4, 7–9, 13, 15, 16, 18, 19, 24, and 25; Figure 1). Of note, the CpG sites previously studied by us, SN8 and SN9 (labeled on Figure 1 by vertical arrow), correspond to CpG19 and CpG8, respectively (20, 21), whereas a frequently used commercially available pyrosequencing methylation assay (PyroMark LINE‐1 kit [Qiagen, Hilden, Germany]) interrogates CpGs 22–24 (labeled in Figure 1 by an “a” marked bracket).

Using this assay, we assessed carcinoma samples from four tissues (bladder, colon, prostate, and stomach) and their matched healthy neighboring tissues; in addition, blood samples were assessed. The strongest methylation changes between tumors and corresponding adjacent tissue were detected in bladder samples; in addition, CpGs at proximal and distal ends of the investigated LINE‐1 region were affected in the prostate; no significant changes were observed in stomach or colon cancers although a clear tendency toward hypomethylation in tumors was observed (Figure 1A, Figure S1). All significant differences resulted from hypomethylation in cancers; however, some CpG sites showed hypermethylation mainly in colon (five CpGs: 2, 9, 15, 16, and 19) and stomach (three CpGs: 9, 15, and 25) tumor samples. The reason why some sites in particular could get hypermethylated is not known.

Having also analyzed neighboring healthy tissues in five tissues, we then asked whether we could identify tissue‐specific methylation patterns. Indeed, based on pairwise comparison of each two tissues, we could identify CpG sites showing different levels of methylation in one specific tissue; the most significant sites were CpG4 in the bladder, CpG19 in the blood, CpG24 in the colon, CpG8 in the prostate, and CpG25 in the stomach (Figure 1B; Figure S2).

Since some CpGs did not show significant variation between the tumor and its neighboring apparently healthy tissue, we sought to explore such invariable/stable CpG sites as internal references/controls. Such an internal reference would allow standardization between different samples, different experiments and different laboratories. Thus, we calculated the ratios of methylation of all combinations of two CpGs. We then compared the ratio of a given CpGs combination between the tumors and the healthy tissues samples (Figure 1C; Figure S3). The best significant ratios were between CpGs 3/19 and 16/25 for the bladder and prostate, respectively (Figure S3). In the case of colon and stomach, this approach was not rewarding as little differences in methylation were detected between the tumors and the neighboring healthy tissues. Overall, this approach did not show good sensitivity to distinguish tumor from healthy tissues, the likely reason, is the tumor‐induced methylation changes in most CpGs and the high number of correlation associated with methylation changes as shown in Figure S1 (right part). Since we have used a global assay, considering locus‐specific methylation in such cases might be more informative.

The mechanisms underlying the heterogeneity of hypomethylation sites within and between tumors are poorly understood, that is, why are not all CpG sites equally hypomethylated and why are not all tumors equally affected? One potential reason could be tissue‐specific signature of potential transcription factor binding sites for LINE‐1. In this context, few transcriptional factor binding sites (YY1, SRY, RUNX3) has already been reported for LINE‐1 (Becker, Swergold, Ozato, & Thayer, 1993; Tchénio, Casella, & Heidmann, 2000; Yang, Zhang, Zhang, & Kazazian, 2003). Undeniably, the contribution of higher‐order chromatin organization and the factors that regulate it cannot be excluded (Fudenberg, Getz, Meyerson, & Mirny, 2011). Alternatively, aberrant methylation profiles of certain tumor‐related genes in urological cancers (bladder cancer and prostate cancer) might have upstream contribution to the LINE‐1 methylation (Florl et al., 2004; Lienert et al., 2011; Wu, Cao, & Wu, 2016). Irrespective of those reasons, our findings imply that the target locus (L1‐CpG sequences) for methylation measurement should be carefully selected as the amount of variation of methylation levels might not be equally detectable at all CpG sites and not equally informative for all tumors. Therefore, our study provides information to overcome this problem. In agreement with our previous work (Nüsgen et al., 2015), we propose that the accuracy to measure this “functional” methylation heterogeneity at L1‐CpG sequences is a key step to develop useful tumor‐tissue‐specific biomarker. To achieve this, the L1‐specific methylation landscape map we have presented here can serve as a guideline to develop more effective methods to study the informative CpG methylation biomarkers in specific tumors.

## 4 CONCLUSION

Our work is the first attempt to dissect the methylation status of each individual CpG site embedded in the LINE‐1 5′‐region. Using a degenerate assay, we have demonstrated that individual CpGs exhibit different levels of methylation and different changes in specific tumors. This observation supports the idea that the reason for discrepancies between various LINE‐1 methylation studies not only relate to the use of different techniques but also to the use of only a few and varying LINE1‐CpG sites, which by default differ in methylation levels. Our data moreover suggest that assays for distinguishing tumor from healthy tissues need to be designed in a more precise tissue‐specific fashion. In this context, our analysis with methylation levels of all technically possible (14 different CpGs sites) which are prone to hypo/hypermethylation will help the researchers to evaluate tumors, neighboring/adjacent healthy tissues and healthy tissue more precisely.

## CONFLICT OF INTEREST

None declared.

## AUTHORS’ CONTRIBUTION

O.E.M. designed the study; A.S. and N.N. performed the experiments; M.A.J. and O.E.M. performed the bioinformatics analysis; A.S., M.A.J., and O.E.M. analyzed the data; A.D., A.G., W.A.S., and J.O. provided the samples, basic contribution to the infrastructure, and discussed the results. A.S. and O.E.M. wrote the manuscript. All authors read and approved the final manuscript.

## ETHICS APPROVAL AND CONSENT TO PARTICIPATE

The study was approved by the Ethics Committee of the Medical Faculty of the HHU and the Lithuanian University Bioethics Committee (approval numbers 1352 and BE‐2‐17, respectively). Written informed consent was obtained from all individuals.

## AVAILABILITY OF DATA AND MATERIAL

Data sharing is not applicable to this article.
