# Supplementary figures and images for: Detailed methylation map of LINE‐1 5′‐promoter region reveals hypomethylated CpG hotspots associated with tumor tissue specificity
Source: Mol Genet Genomic Med. 2019 Apr 6;7(5):e601. doi: 10.1002/mgg3.601 (PMC6503062; doi:10.1002/mgg3.601)

# Supplementary Figure 1

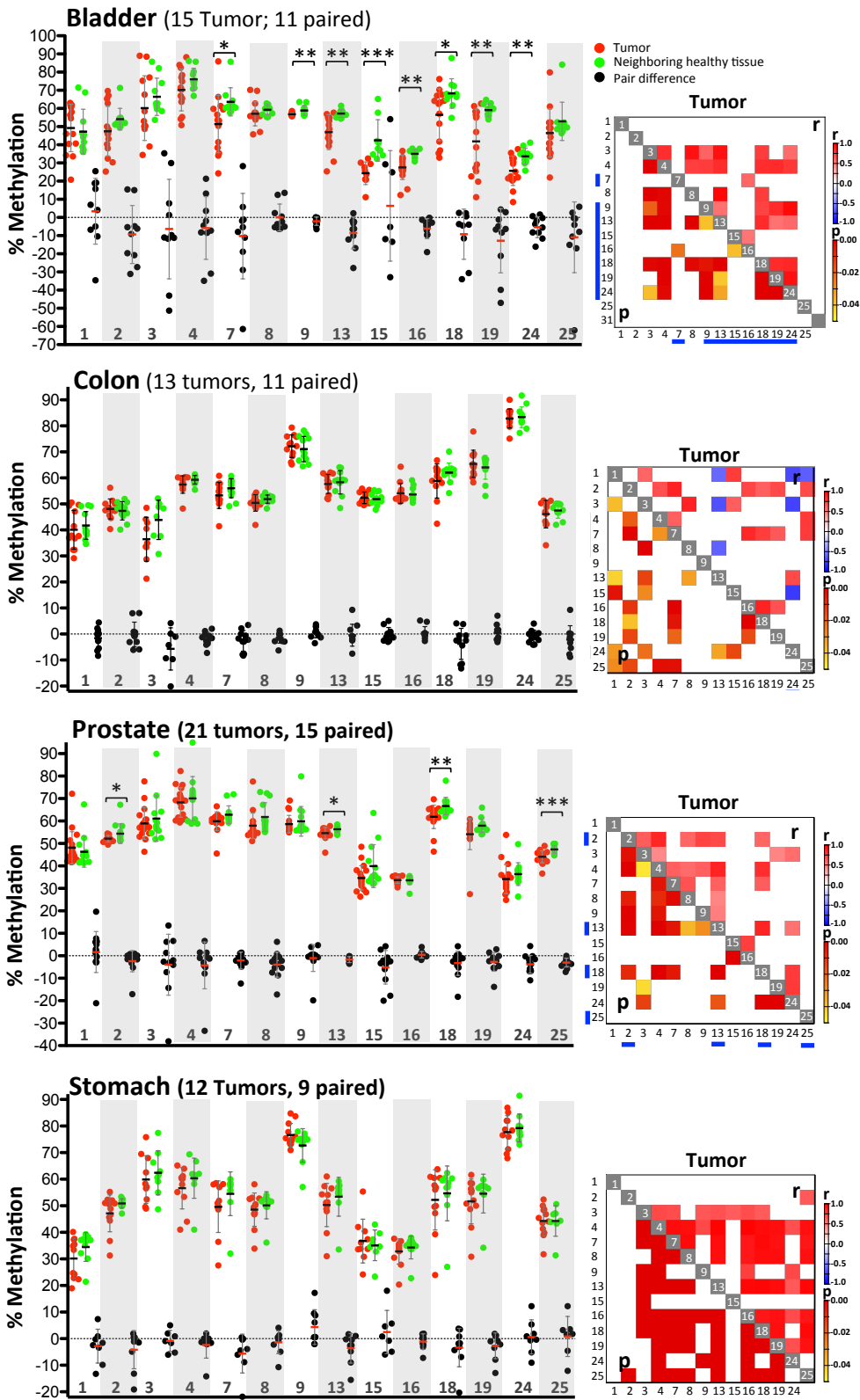

### Supplementary Figure 2

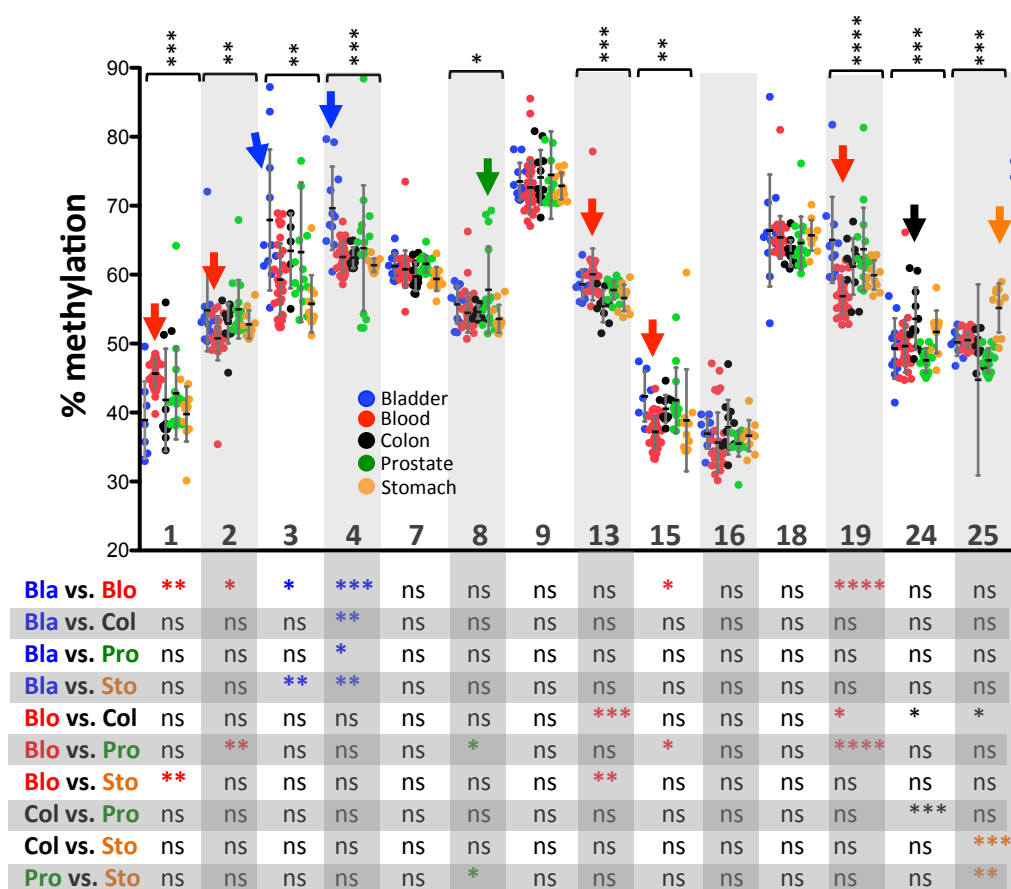

# Supplementary Figure 3

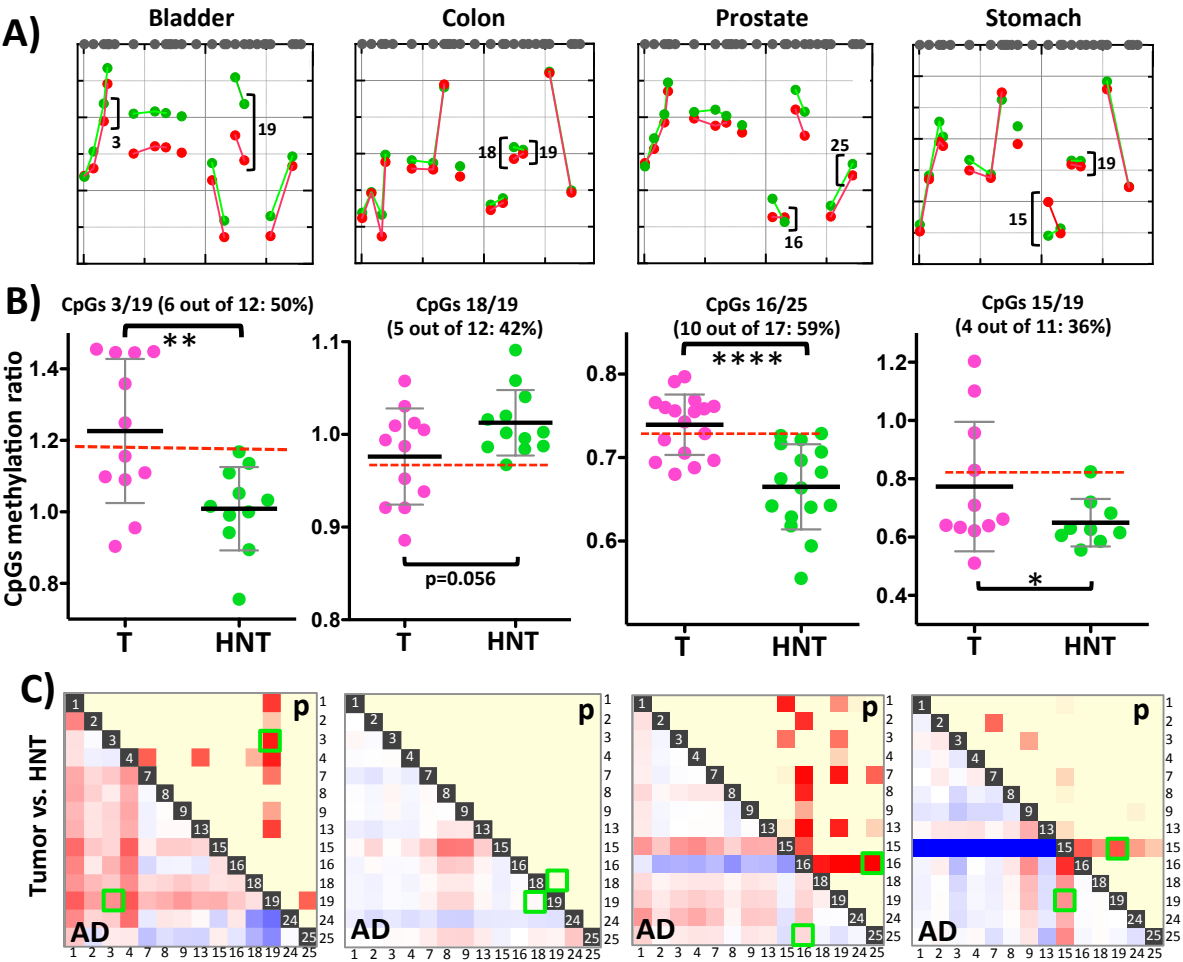

Supplement: Supplementary file 1 [file MGG3-7-e601-s001.pdf]
